# 1-Alkenyl-3-methylimidazolium trifluoromethanesulfonate ionic liquids: novel and low-viscosity ionic liquid electrolytes for dye-sensitized solar cells†

**DOI:** 10.1039/c7ra12904a

**Published:** 2018-04-09

**Authors:** Phuong Tuyet Nguyen, Trang Ngoc Nguyen, Vinh Son Nguyen, Hai Truong Nguyen, Dung Kim Thi Ngo, Phuong Hoang Tran

**Affiliations:** Department of Applied Inorganic Chemistry, Faculty of Chemistry, University of Sciences, Viet Nam National University Ho Chi Minh City 70000 Viet Nam ngtuyetphuong@gmail.com; Department of Organic Chemistry, Faculty of Chemistry, University of Sciences, Viet Nam National University Ho Chi Minh City 70000 Viet Nam thphuong@hcmus.edu.vn

## Abstract

Dye-sensitized Solar Cells (DSCs) based on ruthenium complex N719 as sensitizer have received much attention due to their affordability and high efficiency. However, their best performance is only achieved when using volatile organic solvents as electrolyte solutions, which are unstable under prolonged thermal stress. Thus, we developed a new series of 1-alkenyl-3-methylimidazolium trifluoromethanesulfonate ionic liquids used as robust DSC electrolytes. These ionic liquids exhibit low viscosity, high conductivity, and thermal stability. The implementation of 1-but-3-enyl-3-methyl-imidazolium trifluoromethanesulfonate, [ButMIm]OTf, into DSCs gave the best photovoltaic performance. The results are fairly comparable to those reports for other popular ionic liquid electrolytes currently used in DSC field. An insightful discussion on the relationship between the structure of these new ionic liquids and the *J*–*V* characterization as well as electrochemical impedance measurement of DSCs will give more interesting information. The results are useful for large-scale outdoor application of DSCs.

## Abstract 2

A new series of 1-alkenyl-3-methylimidazolium trifluoromethanesulfonate ionic liquids was prepared under microwave irradiation for application in DSC electrolytes.

## Graphical abstract

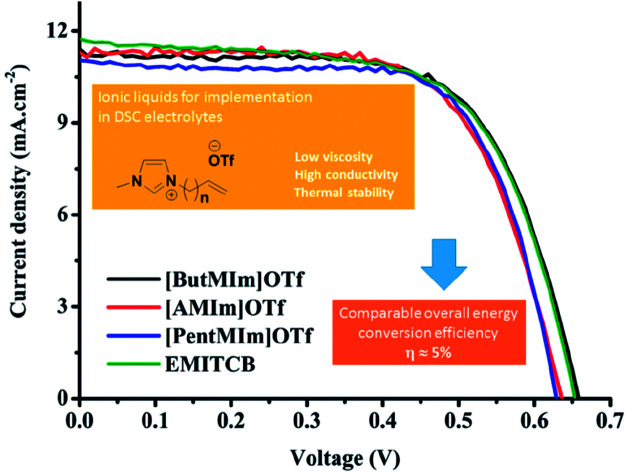

## Introduction

Dye-sensitized solar cells (DSCs) have been developed as promising photovoltaic devices since their first invention by Gratzel and O'Regan.^1–3^ The three basic components constructing a simple DSC are mesoporous metal oxide semiconductor layers deposited on one out of two conductive transparent electrodes, a photosensitizer (or dye) thoroughly loaded onto the mesoporous film, and an electrolyte solution filled up the space between the as-prepared electrode and the other one to assemble a functional DSC. As one of most common electrolytes, the solution of triiodide/iodide redox couple in a high dielectric constant organic solvent is usually employed in high performance DSCs.^2^ An efficiency of 12% has been so far reported as the highest power conversion using this kind of liquid electrolytes in DSC devices applied Ruthenium dyes.^4^ However, high volatility and unavoidable leakage of organic solvents exert a negative impact on the longevity and performance of DSCs.^5,6^ Such so-called “non-robust” electrolytes also caused a dramatically fast thermal degradation of Ruthenium dyes – crucial components in DSCs, leading to a loss of the cell performance.^7,8^ By replacing traditional electrolytes with ionic liquid ones which are known as “robust electrolytes”, thermal stability of the dyes were notably improved to guarantee the passing of serious damn heat test.^9,10^ Hence, more and more researches on the application of ionic liquids as an alternative electrolytes in DSC devices due to their easy synthesis, unique properties, thermal stability, highly ionic conductivity, non-volatility, broad electrochemical potential window, relative non-flammability, and low toxicity have been reported.^11–17^

The ionic liquids are entirely composed of cations (ammonium, phosphonium, guanidinium, pyridinium, and sulfonium) and anions (halides and complex anions such as BF_4_, PF_6_, OTf, NTf_2_, *etc.*) with low melting point below 100 °C.^18–21^ By virtue of this property, ionic liquids have gained widespread applications in DSCs filed as solvents in liquid electrolytes and organic salts in quasi-solid-state electrolytes.^22–27^ 1-Hexyl-3-methylimidazolium iodide was firstly used in DSCs by Papageorgiou's group with the aim of reducing the volatility of electrolyte.^28^ However, the traditional 1-alkyl-3-methylimidazolium iodide is too viscous and the high concentration of iodide preventing them from the practical application of DSCs. Recently, the use of some binary ionic liquid electrolytes allows the reduction of electrolyte viscosity. Wang and coworkers reported a solvent-free electrolyte 1-methyl-3-ethylimidazolium dicyanamide,^29^ 1-ethyl-3-methylimidazolium thiocyanate,^30^ and 1-ethyl-3-methylimidazolium selenocyanate,^31^ and 1-ethyl-3-methylimidazolium tricyanomethanide,^32^ 1-ethyl-3-methylimidazolium tetracyanoborate^33^ blending with standard iodide-based ionic liquids achieved a high conversion efficiency of 6.5 to 8.3%. More latterly, Bidikoudi reported the use of the electrolytes prepared by blending a low viscosity ionic liquid 1-ethyl-3-methylimidazolium dicyanamide with methylimidazolium iodide to enable the devices to attain the efficiencies of 4.4 to 6.5%.^34^ Although these DSCs devices showed the high conversions, the long-term stability was not good. Therefore, the search for alternative electrolytes in DSCs devices has been still studied extensively.

In this paper, we proceed with the synthesis of low-viscosity 1-alkenyl-3-methyimidazolium trifluoromethanesulfonate ionic liquids whose alkenyl chain consists of three to five carbon atoms. The synthesized ionic liquids were structurally identified as well as further characterized for physical properties such as viscosities, conductivities, and thermal stabilities. Then, these low-viscosity ionic liquids were used to prepare electrolytes to be implemented to the functional DSCs. The cells were finally characterized by *J*–*V* curve and electrochemical impedance measurement.

## Result and discussion

**Scheme 1:**
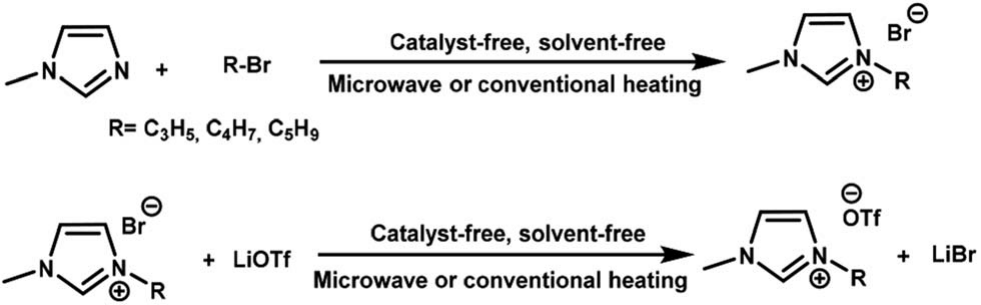
Synthesis of 1-alkenyl-3-methylimidazolium trifluoromethanesulfonates.

The low-viscosity ionic liquids were easily prepared in high yields *via* a two-step pathway procedure under solvent- and catalyst-free condition assisted by microwave irradiation or conventional heating. The alkylation of imidazole by alkyl bromides followed by anion metathesis with lithium triflate resulted in the formation of three new ionic liquids, 1-allyl-3-methylimidazolium trifluoromethanesulfonate [AMIm]OTf, 1-but-3-enyl-3-methyl-imidazolium trifluoromethanesulfonate [ButMIm]OTf, and 1-pent-4-enyl-3-methylimidazolium trifluoromethanesulfonate [PentMIm]OTf (Scheme 1).

The results from Table 1 showed that the ionic liquids were obtained in excellent yield (68–90%) with high purity. Compared to conventional heating, microwave irradiation was a better method to afford the ionic liquids in higher yields.

**Table 1:** The preparation of 1-alkenyl-3-methylimidazolium trifluoromethanesulfonates under solvent-free conditions

Entry	ILs	Condition (°C, min)		Yield^a^ (%)	
		Step 1	Step 2	MW^b^	*Δ^c^*
1	[AMIm]OTf	100, 20	100, 15	90	78
2	[ButMIm]OTf	100, 20	100, 15	88	72
3	[PentMIm]OTf	100, 20	100, 30	89	68

a Isolated yield.

b Microwave heating: the reaction was performed in closed vessels using a CEM Discover monomode oven with strict control of pressure and temperature (power 10 W).

c Conventional heating: the reaction was performed in a thermostat-controlled oil bath.

The fact that ionic liquids are more viscous than traditional organic solvents can impede them from being used as electrolytes in DSCs. Fortunately, the viscosity of a given ionic liquid can be greatly governed by the nature of its anion as reported by Willner.^35^ For example, the replacement of three fluorine atoms in the [PF_6_] anion by pentafluoroethyl groups tremendously decreases the viscosity of imidazolium ionic liquids from 548 to 74 cP.^35^ Consequently, we developed a new series of low-viscosity imidazolium triflate ionic liquids bearing different alkenyl substituents. As expected, the replacement of halide anions with triflate anion reduced the viscosity of imidazolium ionic liquids remarkably. The viscosity of synthesized ionic liquids was given in Table 2. As reported by Chen and coworkers, the viscosity values of a range of ILs having the same cation but different anions increase in the order: [DCA] < [NTf_2_] < [TfO] < [BF_4_] < [PF_6_] < [OAc].^36^ For example, the value of viscosity for [BMIm][OTf] is 75 cP, whereas it is only 45 cP for [BMIm][NTf_2_].^36^ In the current work, we found that the replacement of alkyl substituents by alkenyl substituents led to decrease the viscosity of ionic liquids. The viscosity of [ButMIm][OTf] is only 16.9 cP (Table 2). Generally, the viscosity of ionic liquids increases with the length of alkyl side chain due to the increase of the van der Waals interaction.^36^ However, there is somewhat different in the viscosity of [AMIm][OTf]/33.7 cP > [ButMim][OTf]/16.9 cP presumably due to a more flexibility of butenyl than allyl.

**Table 2:** Viscosity and conductivity of 1-alkenyl-3-methylimidazolium trifluoromethanesulfonates

	[AMIm]OTf	[ButMIm]OTf	[PentMIm]OTf
Viscosity (cPs)	33.7	16.9	94.4
Conductivity (mS cm^−1^)	3.96	12.13	0.68

**Fig. 1:**
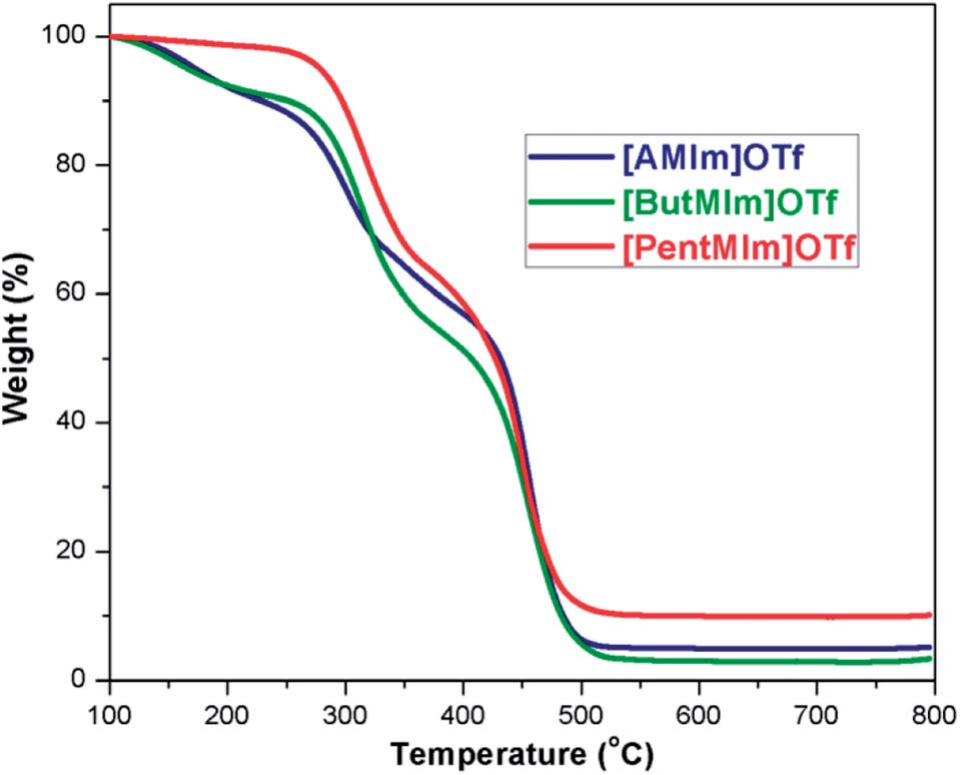
TGA curves of [AMIm]OTf, [ButMIm]OTf, and [PentMIm]OTf.

Thermal gravimetric analyses (TGA) of [AMIm]OTf, [ButMIm]OTf, [PentMIm]OTf were conducted to test their thermal stability and the results were presented in Fig. 1. The analysis shows that the 1-alkenyl-3-methylimidazolium trifluoromethanesulfonate ionic liquids are stable at high temperature (up to 180 °C, 200 °C, and 280 °C for [AMIm]OTf, [ButMIm]OTf, [PentMIm]OTf, respectively), which ensure these ionic liquids suitable as the electrolyte in DSCs.

**Fig. 2:**
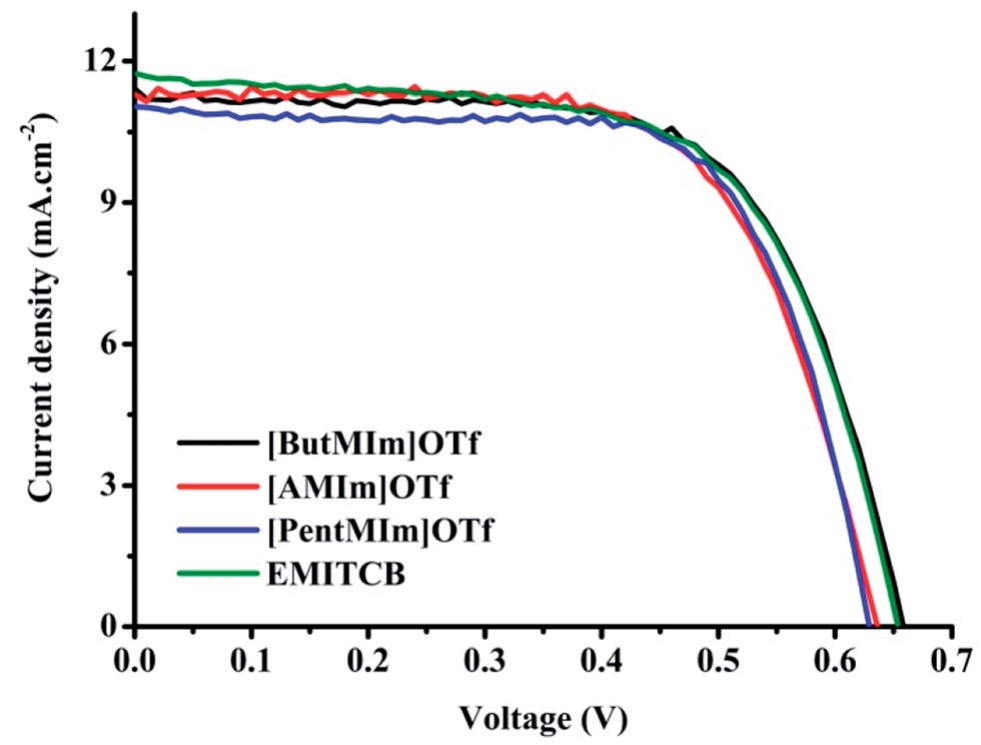
Typical *J*–*V* curves of DSCs using ionic liquid electrolytes with [PentMIm]OTf (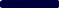); [ButMIm]OTf (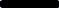); [AMIm]OTf (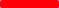) and EMITCB (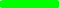) under the light intensity of 1000 W m^−2^. The electrolyte composition: 0.05 M I_2_, 0.1 M PMII, 0.6 M GuNCS, 0.5 M NBB, and either one of these four ionic liquids.

Fig. 2 shows current–voltage *J*–*V* characteristics of the cells using ionic liquid electrolytes mixed with PMII in comparison with the popular one 1-ethyl-3-methylimidazolium tetracyanoborate (EMITCB) under AM 1.5G illumination. The respectively photovoltaic parameters are involved in Table 3. In general, all the *J*–*V* curves show similar shape in all three cases of synthesized ionic liquid electrolytes. The open circuit voltage (*V*_OC_) of DSC with [ButMIm]OTf is equivalent to EMITCB, and higher than the ones with two other ionic liquids about 20–30 mV while the short circuit current density (*J*_SC_) values are just slightly different between the three ionic liquid electrolytes.

**Table 3:** Photovoltaic performance of DSCs used the synthesized ionic liquid electrolytes in comparison to the popular one EMITCB under the light intensity of 1000 W m^−2^. Bold values are selected for *J*–*V* characteristics illustration in Fig. 2

Ionic liquid	*J*_SC_, mA cm^−2^	*V*_OC_, V	FF	Efficiency, %
**[ButMlm]OTf**	**11.413**	**0.659**	**0.653**	**4.907**
	11.115	0.652	0.673	4.876
	11.311	0.653	0.655	4.833
**[AMIm]OTf**	**11.660**	**0.626**	**0.694**	**5.067**
	11.560	0.624	0.684	4.933
**[PentMlm]OTf**	10.820	0.631	0.692	4.727
	**11.042**	**0.630**	**0.693**	**4.820**
	10.828	0.632	0.701	4.791

As can be seen in Table 3 there were no considerable differences in the photovoltaic parameters among the cells belonging to this electrolytes series. The photovoltaic performance of DSC with the synthesized ionic liquids is comparable to DSC with the popular EMITCB. Cells using [PentMIm]OTf and [AMIm]OTf are almost the same in *V*_OC_ (0.63 V in average) which is slightly lower than cells using [ButMIm]OTf. However, the fill factor (FF) values with these two ionic liquid electrolytes are about 69–70% and higher than [ButMIm]OTf (∼65–66%). The cells with [PentMIm]OTf have the lowest in *J*_SC_ values, therefore the lowest in the overall efficiency energy conversion which is about 4.8% compared to 4.9% and 5.0% with [ButMIm]OTf and [AMIm]OTf, respectively. The viscosity and ionic conductivity can be explained as the main factors responsible for these differences. The results of photovoltaic performance correspond reasonably with the viscosity and conductivity values of our ionic liquids in which [ButMIm]OTf has the lowest viscosity (16.9 cPs) and the highest conductivity (12.13 mS cm^−1^), thus leading to the best voltage and current. Further data summarized in Table S1 (ESI†) shows that DSCs with our ionic liquids are well comparable to the results reported elsewhere with different ionic liquids.

**Fig. 3:**
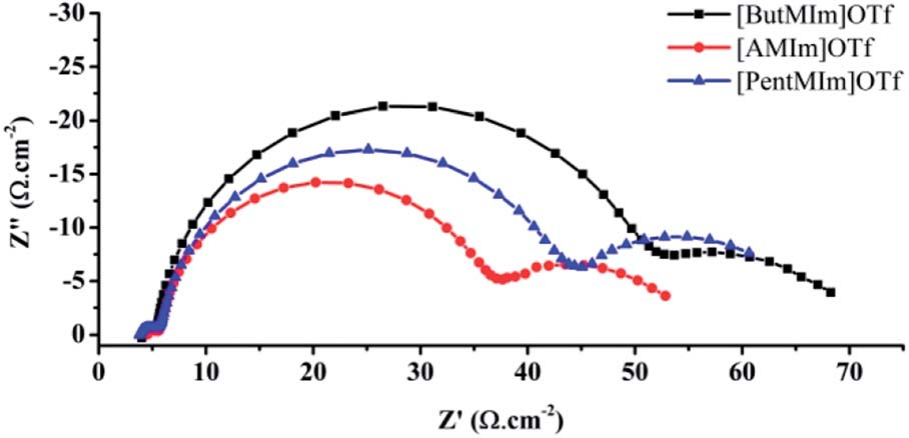
Nyquist plots showing impedance data of DSCs using trifluoromethanesulfonate electrolytes. The data were obtained at an applied potential of 0.60 V. The solid lines are model fit to the data.

Fig. 3 shows an analysis of the electrochemical impedance spectroscopy (EIS) of the DSCs using the synthesized ionic liquid electrolytes. The measurement was performed in the dark under a forward bias of −0.60 V. As shown in Fig. 3; three arcs can be observed in the Nyquist plots in the frequency range of 0.01 Hz to 100 kHz. The first arc at the high-frequency range (10^3^ to 10^5^ Hz) is related to the charge transfer processes at counter electrode|electrolyte interface, the second arc at the middle frequency range (1–10^3^ Hz) is related to carrier transport resistance (*R*_CT_) in TiO_2_|dye|electrolyte interfaces, and the final one at the low-frequency range (2 × 10^−2^ to 1 Hz) is associated with ionic diffusion in the electrolyte.

As can be seen from Fig. 3, the charge transfer resistant *R*_CT_ are about 43, 35, 29 Ω cm^−2^ for the case of using [ButMIm]OTf, [PentMIm]OTf and [AMIm]OTf, respectively, indicating that the back electron transfer at the interface between TiO_2_ photo anodes and electrolytes increases within the order of the mentioned series ionic liquids. Therefore, DSC with [ButMIm]Otf responds the high performance in both voltage and current. Differently, *R*_CT_ of cells using [PentMIm]OTf is observed to be higher than the ones with [AMIm]OTf while the *J*_SC_ values show a completely opposite tendency. This can be explained by the effects of other parameters as charge transport resistance *R*_t_; capacitance and electron life time of the DSCs which will be discussed further below.

The third arc related to ionic electrolyte diffusion can be evaluated by the shape of the arc and diffusion resistant value *R*_D_ obtained from it. The shapes of the third arcs with [PentMIm]OTf and [AMIm]OTf are similarly whereas the one with [ButMIm]OTf is different in the slope of the left side upward of the arc. This illustrates that with [ButMIm]OTf the electrolyte performs better ionic diffusion, and there is less electron transfer between the interaction of electrolyte and the two electrodes. The values of *R*_D_ were obtained to be 18, 22, 16 Ω cm^−2^ for [ButMIm]OTf, [PentMIm]OTf and [AMIm]OTf, respectively.

From the calculation and simulated data, the values of *R*_t_, *R*_CT_ and capacitance were extracted. We also obtained further analysis on Bode plots. From the Bode plots, the electron life times (*τ*) of these cells were estimated using eqn (1), where the *f*_max_ value was read off the Bode plot at the phase angle peak observed from the second peak.1
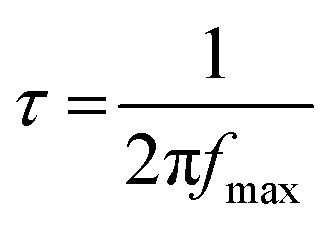


**Fig. 4:**
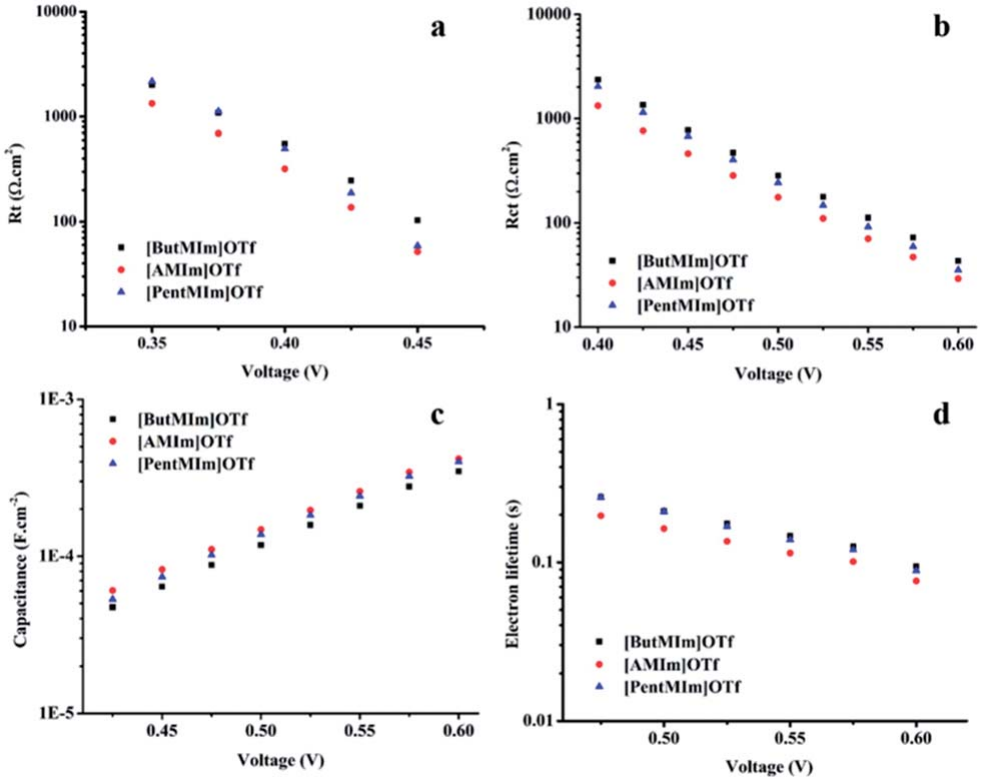
Charge electron transfer resistant, *R*_CT_ (a); charge transport resistant, *R*_t_ (b); capacitance (c) and electron life time, *τ* (d) of the DSCs using 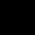 [ButMIm]OTf, 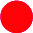 [AMIm]OTf, 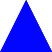 [PentMIm]OTf calculated from simulated data and the Bode plots. Data was collected at various applied potentials.

Fig. 4 shows linear curves of *R*_CT_; *R*_t_; capacitance; as well as *τ* values when the applied potentials increase in all the three ionic liquids, indicating that all the cells respond with the same behaviour at different potentials. The data again confirm the less back electron transfer between electrolyte mediators and photo-anodes, as well as better electrolyte ionic diffusion, leading to the best photovoltaic performance with [ButMIm]OTf electrolyte. These results are well-agreed with the low viscosity and good conductivity values of [ButMIm]OTf. Whereas the cases of [AMIm]OTf and [PentMIm]OTf are more complicated. The ionic liquid [AMIm]OTf owns lower viscosity and a bit higher conductivity than [PentMIm]OTf, so its DSCs give better current and voltage. However, the *R*_CT_ values of DSCs with [AMIm]OTf are lower than [PentMIm]OTf as aforementioned. This can be explained by the contribution of other parameters including *R*_t_; capacitance and *τ* to the current value. Fig. 4b and c show that DSCs with [AMIm]OTf have higher capacitance, and especially lower charge transport resistant *R*_t_ than [PentMIm]OTf. The difference between these values *R*_CT_; *R*_t_; and capacitance might be due to their dissimilar structure. The short alkenyl chain might not somehow help to protect the interaction between electrolytes and electrodes, therefore DSCs with [AMIm]OTf have low *R*_CT_, hence, less efficient electron life time than DSCs with [PentMIm]OTf. But the viscosity and conductivity of the ionic liquids have more impact to the cell performance, therefore it gives a finally higher overall energy conversion in the case of [AMIm]OTf.

## Experimental section

### General procedure for the preparation of 1-allyl-3-methylimidazolium trifluoromethanesulfonate ([AMIm]OTf) under microwave irradiation

In a 5 mL round-bottom flask, a mixture of 1-methylimidazole (2 mmol, 0.1640 g) and allyl bromide (2 mmol, 0.2409 g) was irradiated under microwave heating at 100 °C for 20 min. Then, LiOTf (2 mmol, 0.3120 g) was added and the resulting mixtures were further irradiated at 100 °C for 15 min. The reaction mixture was cooled to ambient temperature, and then diluted with 5 mL of acetonitrile. After filtration through celite, the solvent was removed. The crude products were washed with diethyl ether and concentrated by rotary evaporator to obtain [AMIm]OTf whose structure was confirmed by ^1^H, ^13^C NMR spectroscopy, and HRMS (ESI).

### DSCs fabrication and characterization

The dye-sensitized solar cells were constructed by two electrodes prepared from glass substrates (Pilkington −8 Ω cm^−2^) coated with F-doped SnO_2_ (FTO) as previously described in our publications elsewhere.^37,38^ DSC electrolyte was a mixture of 0.05 M I_2_, 0.1 M PMII, 0.6 M GuNCS, 0.5 M NBB, and either one of four ionic liquids including 1-ethyl-3-methylimidazolium tetracyanoborate (EMITCB) and the three synthesized ionic liquids [AMIm]OTf or [ButMIm]OTf, or [PentMIm]OTf. Each experiment produced 8 to 10 DSCs.

Photovoltaic measurements of DSCs were performed following the protocol as described^37^ and the DSCs were masked with a 0.1444 cm^2^ active area of the anode electrode.

Electro impedance spectroscopy (EIS) was carried out using an electrochemical interface workstation (Schlumberger SI-1286) and a HF frequency response analyzer (Schlumberger SI-1255). Different bias potentials from −0.3 V to open-circuit voltage value, synchronized with a modulated voltage of 10 mV with a frequency range of 100 kHz to 10 MHz, were applied in the dark. The data was analyzed by using Z-View software with the appropriate equivalent circuit.

## Conclusions

Three low-viscosity ionic liquids, based on 1-alkenyl-3-methyimidazolium cations and trifluoromethane (OTf-) anion, where the alkenyl chain length was 3 to 5 carbon atoms, were synthesized and implemented in DSC electrolytes. [ButMIm]OTf shows the lowest viscosity although its structure does not have the shortest alkenyl chain. The solar cells using [ButMIm]OTf in electrolytes also respond the best photovoltaic performance due to its best conductivity and low viscosity. The cells using [AMIm]OTf were obtained better short circuit current values than the ones with [PentMIm]OTf. All the DSCs applied these three ionic liquids show comparable performance to the present popular ones demonstrating their high potential for the use in DSCs. For further application of these ionic liquids in DSC devices, the long term stability of DSCs with these ionic liquid electrolytes should be investigated.

## Conflicts of interest

There are no conflicts to declare.

## Supplementary Material

RA-008-C7RA12904A-s001
