# Supplementary material for: 1-Alkenyl-3-methylimidazolium trifluoromethanesulfonate ionic liquids: novel and low-viscosity ionic liquid electrolytes for dye-sensitized solar cells
Source: RSC Adv. 2018 Apr 9;8(24):13142–7. doi: 10.1039/c7ra12904a (PMC9079676; doi:10.1039/c7ra12904a)
Supplement: RA-008-C7RA12904A-s001 [file RA-008-C7RA12904A-s001.pdf]

Electronic Supplementary Material (ESI) for RSC Advances.
This journal is © The Royal Society of Chemistry 2018

Supporting information

For

1-Alkenyl-3-methylimidazolium trifluoromethanesulfonate ionic liquids: novel and low-viscosity ionic liquid electrolytes for dye-sensitized solar cells.

Phuong Tuyet Nguyen[a*], Trang Ngoc Nguyen[a], Vinh Son Nguyen[a], Hai Truong Nguyen[b], Dung Kim Thi Ngo[b] and Phuong Hoang Tran[b*]

[a]Department of Applied Inorganic Chemistry, Faculty of Chemistry, University of Sciences, Viet Nam National University, Ho Chi Minh City 70000, Viet Nam.

[b]Department of Organic Chemistry, Faculty of Chemistry, University of Sciences, Viet Nam National University, Ho Chi Minh City 70000, Viet Nam.

Corresponding author: ntphuong@hcmus.edu.vn (Phuong Tuyet Nguyen), thphuong@hcmus.edu.vn (Phuong Hoang Tran)

Table of contents

Section S1. Syntheses of materials **S1**

Section S2. The comparison for photovoltaic performance of DSCs applied different types of electrolyte solutions based on ionic liquids **S2-S3**

Section S3. Spectral data **S3-S16**

Section S4. References **S17**

Section S1. Syntheses of Materials

Chemicals and supplies.

1-Methylimidazole (*ReagentPlus*®, 99%), Allyl bromide (reagent grade, 97%), 4-bromo-1-butene (assay, 97%), 5-bromo-1-pentene (assay, 95%), lithium trifluoromethansulfonate (assay, 96%), iodomethane (grade analytical standard), iodoethane (assay, 99%), 1-iodopropane (assay, 99%) were obtained from Sigma-Aldrich. Celite was obtained from Merck. Acetonitrile (purity ≥ 99.5%), diethyl ether (≥ 95%), chloroform (purity ≥ 99%) were obtained from Xilong Chemical Co., Ltd (China). Chloroform-*d*, 99.8 Atom %D, stab. with Ag was obtained from Armar (Switzerland).

All starting materials, reagent and solvents were used without further purification.

Analytical techniques.

The ^{1}H and ^{13}C NMR spectra were recorded on a Bruker Advance 500 instruments using $CDCl_3$ as solvent and solvent peaks or TMS as internal standards. Thermal gravimetric analysis (TGA) was measured on a TA Q500 thermal analysis system with the sample held in a platinum pan in a continuous airflow. Vicosity of ionic liquids was performed using Brookfield DV-III programmable Rheometer (at room temperature ~30 ^{o}C). The ionic conductivity of ILs was measured by using Conductimeter OAKION CON 2700. HRMS (ESI) data were recorded on Bruker micrOTOF-QII MS at 80 eV.

Section S2. The comparison for photovoltaic performance of DSCs applied different types of electrolyte solutions based on ionic liquids

Table S1. Photovoltaic performance of DSCs applied different types of electrolyte solutions based on ionic liquids.

Entry	Composition of IL electrolyte	J_{SC} (mA/cm^2)	V_{OC} (V)	Fill factor	Efficiency (%)	Light Intensity (mW/cm^2)	Dye
1	10% HMImI, 90% EMImTf$_2$N, 5 mM I_2[1]	11.8	0.57	0.72	-	100	N-3
	10% HMImI, 90% EMImTfO, 5mM I_2[1]	9.5	0.23	0.69	-	100	N-3
2	0.9M DMHImI, 30 mM I_2 in EMImTFSI[2]	0.9	0.50	0.80	0.36	100	N-3
	0.9M DMHImI, 30 mM I_2 in EMImF·2.3HF[2]	5.8	0.65	0.56	2.1	100	N-3
3	0.1M I_2, 0.45M NMBI in PMII and EMIDCN (13:7 v/v)[3]	10.4	0.74	0.74	5.7	100	Z-907
	0.1M I_2, 0.1M LiI, 0.45M NMBI in PMII and EMIDCN (13:7 v/v)[3]	12.8	0.71	0.73	6.6	100	Z-907
	0.1M I_2, 0.1M LiI, 0.45M NMBI in PMII and EMIDCN (13:7 v/v)[3]	-	0.68	-	5.0	100	N-719
4	0.2M I_2, 0.14M GuSCN, 0.5M TBP in PMII and EMINCS (13:7 v/v)[4]	11.4	0.74	0.76	6.4	100	Z-907
5	[I^-:I_2=10:1], [I^-+I_3^-]=2M, 1M TBP, 0.5M LiI in EMImDCA[5]	11.8	0.73	0.63	5.5	100	N-3
	[I^-:I_2=10:1], [I^-+I_3^-]=1.5M, 1M TBP, 0.1M LiI in EMImTFSI[5]	12	0.63	0.59	4.5	100	N-3
6	I_2, 0.5M NMBI in PMII and EMImTCM (1:1 v/v)[6]	12.81	0.75	0.76	7.4	100	Z-907Na
7	0.2M I_2, 0.5M NMBI, 0.1M GuSCN in PMII and EMIB(CN)$_4$ (13:7 v/v)[7]	12.7	0.72	0.70	6.4	100	Z-907Na
8	0.2M I_2, 0.1M GuSCN, 0.5M NMBI in PMII and EMIB(CN)$_4$ (65:35 v/v)[8]	14.56	0.71	0.70	7.2	100	K-77
9	0.8M PMII, 0.1M PMIIBr$_2$, 0.1M GuSCN, 0.5M NBMI in ɣ-butyrolactone[9]	1.3	0.75	0.73	7.3	100	N-3
10	0.6M MVII, 0.06M I_2, 0.6M LiI, 0.5M TBP in propylene carbonate[10]	12.19	0.67	0.61	4.98	100	N-3
11	(Bu$_2$MeS)I:I_2=100:1[11]	0.80	0.55	0.52	2.3	100	N-719
12	0.15M I_2, 0.5M TBP, 0.1M GuSCN, PMII and EMIMBF$_4$ (1:1 v/v)[12]	13.67	0.63	0.58	4.99	100	N-3
13	0.3M I_2, 1.5M PMII, 0.1M LiI, 0.5M TBP in S$_{53}$TFSI[13]	8.28	0.64	0.60	3.27	100	N-719
14	0.03M I_2, 0.1M GuSCN, 0.5M TBP, 0.6M FIL in acetonitrile and valeronitrile (85:15 v/v)[14]	11.50	0.61	0.73	5.1	30	Z-907

15	T_2:EMITCM:PMIT=2:5.6:10, T_2=0.64M[15]	10.71	0.66	0.47	3.30	100	N-719
16	2M I_2, 2M LiI, PMII and BMISCN (1:0.75 v/v)[16]	6.52	0.62	0.47	1.89	100	N-719
17	0.05M I_2, 0.1M LiI, 0.6M butyl substituted imidazolium iodine salt[17]	17.60	0.60	0.49	5.17	100	N-719
18	Our work: 0.05 M I_2, 0.1 M PMII, 0.6 M GuNCS, 0.5 M NBB, and [ButMIm]OTf	11.41	0.66	0.65	4.91	100	N719

Section S3. Spectral data

1-Allyl-3-methylimidazolium tetrafluoromethanesulfonate [AMIm](OTf)

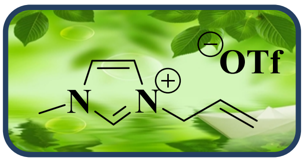

^{1}H NMR (500 MHz, $CDCl_3$) δ 9.62 (s, 1H), 7.42 (s, 1H), 7.33 (s, 1H), 6.01–5.95 (q, J = 10.5 Hz, 17.0 Hz, 1H), 5.46–5.42 (t, J = 8.5 Hz, 2H), 4.88–4.87 (d, J = 6.5 Hz, 2H), 4.00 (s, 3H).

^{13}C NMR (125 MHz, $CDCl_3$) δ 135.9, 127.8, 121.6, 121.0, 119.8, 114.5, 50.3, 34.9.

HRMS (ESI) *m/z* Calcd for $[M]^+$123.0927; Found 123.0971.

Viscosity (η) (~30 °C): 33.7 cP.

Conductivity (σ_c) (~30 °C): 0.780 (mS.cm^{-1}).

1-Butenyl-3-methylimidazolium tetrafluoromethanesulfonate [ButMIm](OTf)

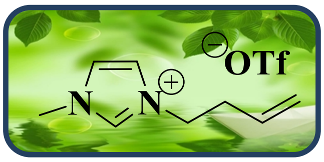

^{1}H NMR (500 MHz, $CDCl_3$) δ 9.82 (s, 1H), 7.31 (s, 2H), 5.81 – 5.73 (m, 1H), 5.13 – 5.07 (t, J = 10.0 Hz, 2H), 4.38 – 4.35 (t, J = 7.0 Hz, 2H), 4.02 (s, 3H), 3.85 (s, 1H), 3.73 (s, 1H).

^{13}C NMR (125 MHz, $CDCl_3$) δ 137.2, 132.2, 123.4, 122.2, 119.6, 116.4, 49.2, 36.5, 34.2.

HRMS (ESI) *m/z* Calcd for $[M]^+$ 137.1073; Found 137.1159.

Viscosity (η) (~30 °C): 16.9 cP.

Conductivity (σ_c) (~30 ^{o}C): 0.618 (mS.cm^{-1}).

1-Pentenyl-3-methylimidazolium tetrafluoromethanesulfonate [PentMIm](OTf)

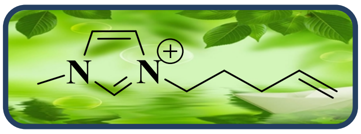

^{1}H NMR (500 MHz, $CDCl_3$) δ 9.88 (s, 1H), 7.34 (s, 1H), 7.44 (s, 1H), 5.70 – 5.62 (m, 2H), 4.96 – 4.89 (q, J = 17.0 Hz, 8.0 Hz, 2H), 4.23 – 4.20 (t, J = 2.0 Hz, 1H), 3.98 (s, 3H), 1.95 – 1.92 (m, 4H).

^{13}C-NMR (125 MHz, $CDCl_3$) δ 136.9, 136.0, 123.8, 122.2, 116.3, 49.3, 36.6, 30.0, 29.1.

HRMS (ESI) *m/z* Calcd for $[M]^+$ 151.1230 ; Found 151.1221.

Viscosity (η) (~30 ^{o}C): 94.4 cP.

Conductivity (σ_c) (~30 ^{o}C): 12.910 (mS.cm^{-1}).

^{1}H NMR, ^{13}C NMR, HRMS, viscosity of [AMIm]OTf

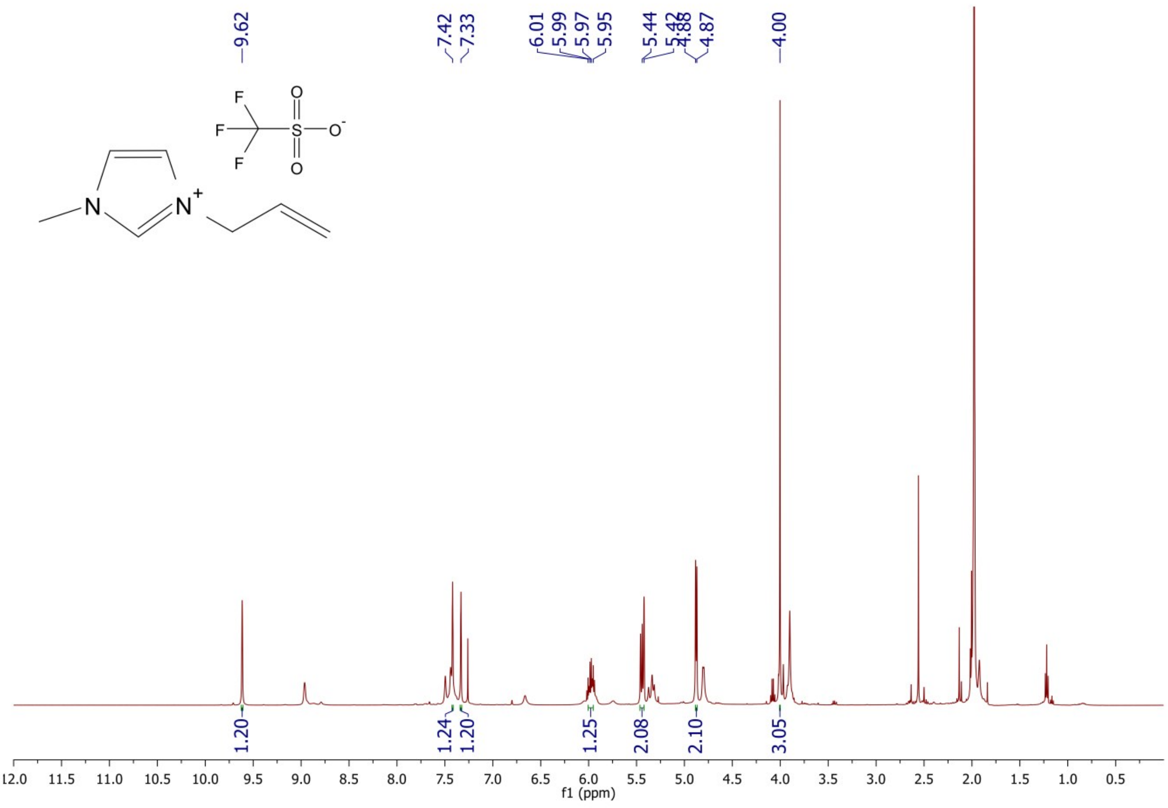

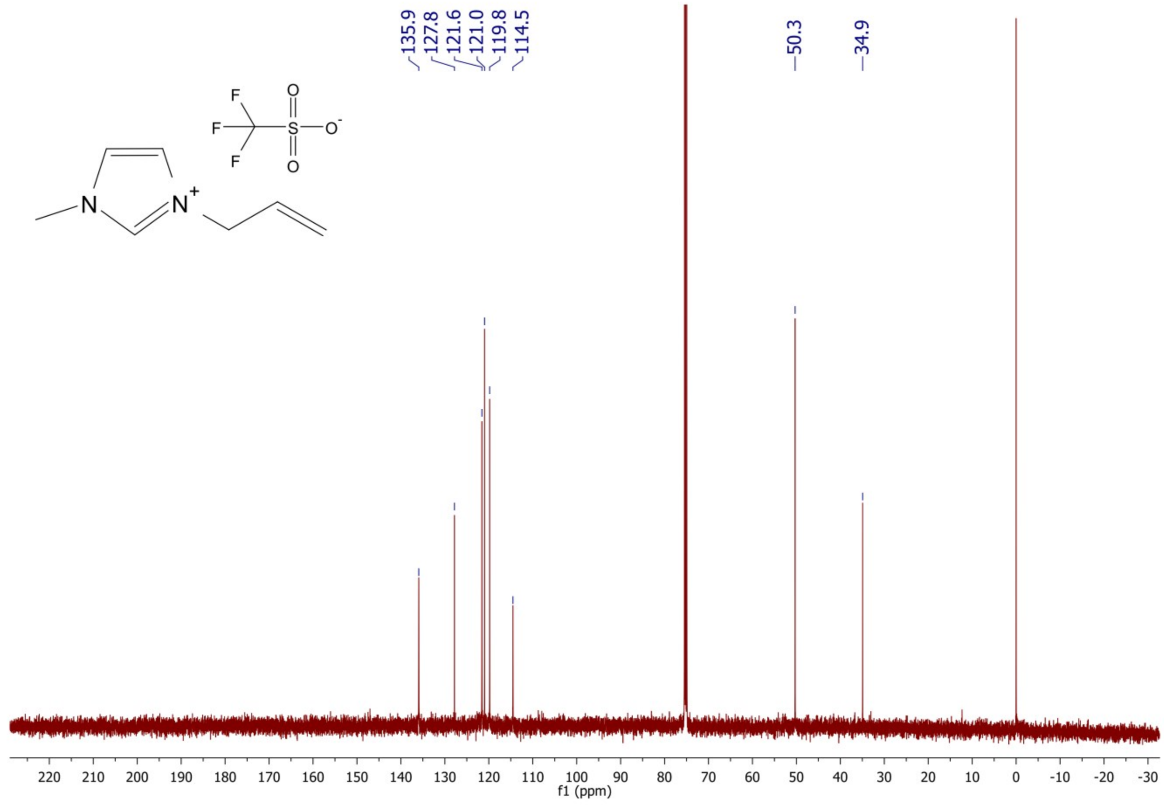

Generated by Foxit PDF Creator © Foxit Software
http://www.foxitsoftware.com For evaluation only.

Display Report

Analysis Info

Analysis Name	D:\Data\dmm 2015\AMIM _1-F,2_01_16187.d	Acquisition Date	5/25/2015 9:39:53 PM
Method	dmm.m	Operator	Mai
Sample Name	AMIM	Instrument	micrOTOF-Q 10187
Comment			

Acquisition Parameter

Source Type	ESI	Ion Polarity	Positive	Set Nebulizer	2.0 Bar		
Focus	Not active	Set Capillary	4500 V	Set Dry Heater	200 °C		
Scan Begin	100 m/z	Set End Plate Offset	-500 V	Set Dry Gas	9.0 l/min		
Scan End	3000 m/z	Set Collision Cell RF	250.0 Vpp	Set Divert Valve	Source		

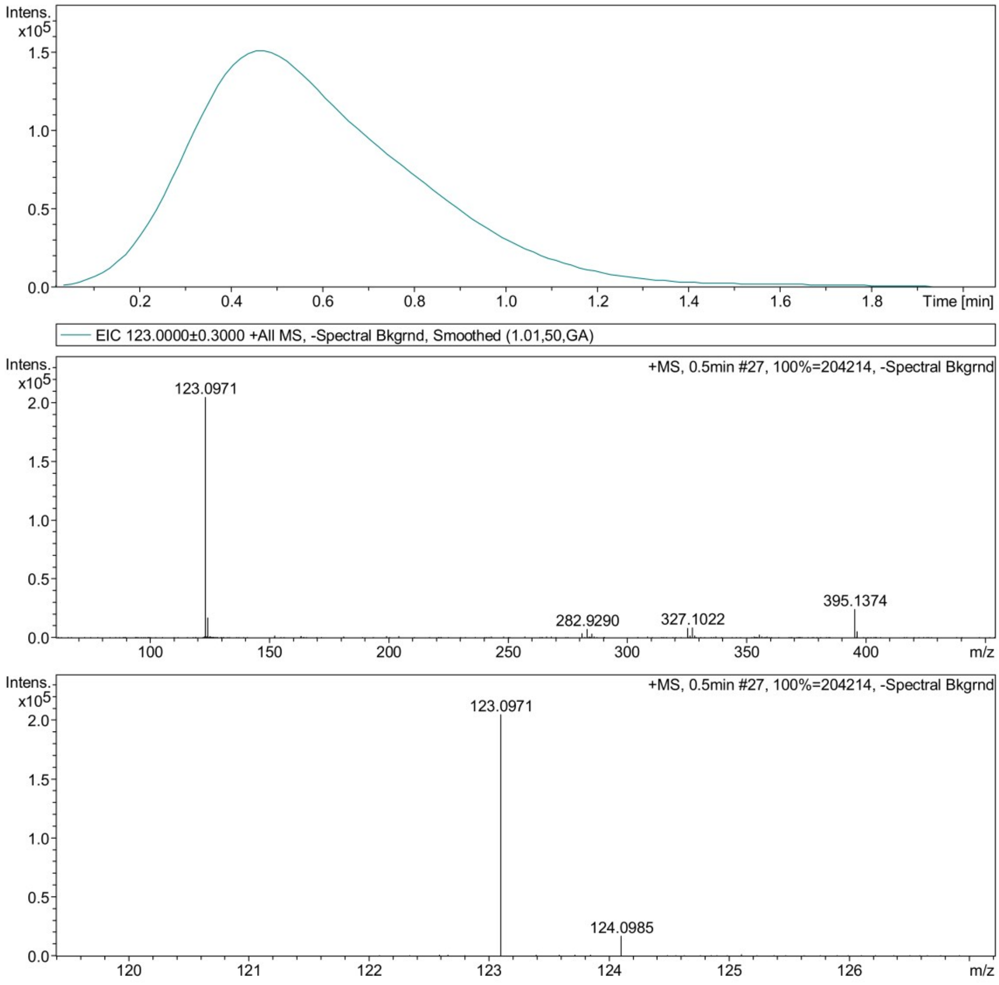

Rheocalc V2.7 **Brookfield Engineering Labs**

Math Model: Bingham

Plastic Viscosity: 33,7 cP **Yield Stress: -2,51 D/cm²**

Confidence of Fit: 95,0 %

File: F:\24-6-15\AMIM.DB

Test Date: 25/06/2015 **Test Time:** 7:48:16 SA **Model:** RV **Spindle:** CP40

Sample Name: AMim

#	Viscosity (cP)	Speed (RPM)	% Torque (%)	Shear Stress (D/cm²)	Shear Rate (1/sec)	Temperature (°C)	Time Interval (mm:ss.t)
1	34,01	5,00	5,2	12,75	37,50	30,1	00:30,
2	35,97	10,00	11,0	26,98	75,00	30,0	00:30,
3	30,96	15,00	14,2	34,83	112,50	30,0	00:30,
4	30,41	20,00	18,6	45,62	150,00	30,0	00:30,
5	30,61	25,00	23,4	57,39	187,50	30,2	00:30,
6	31,39	30,00	28,8	70,63	225,00	30,1	00:30,
7	32,05	35,00	34,3	84,12	262,50	30,0	00:30,
8	32,78	40,00	40,1	98,35	300,00	30,1	00:30,
9	33,06	45,00	45,5	111,59	337,50	30,2	00:30,
10	33,68	50,00	51,5	126,30	375,00	30,2	00:30,
11	33,53	55,00	56,4	138,32	412,50	30,0	00:30,
12	33,35	60,00	61,2	150,09	450,00	30,2	00:30,

Notes:

Page 1 **25/06/2015 8:03:03:39 SA**

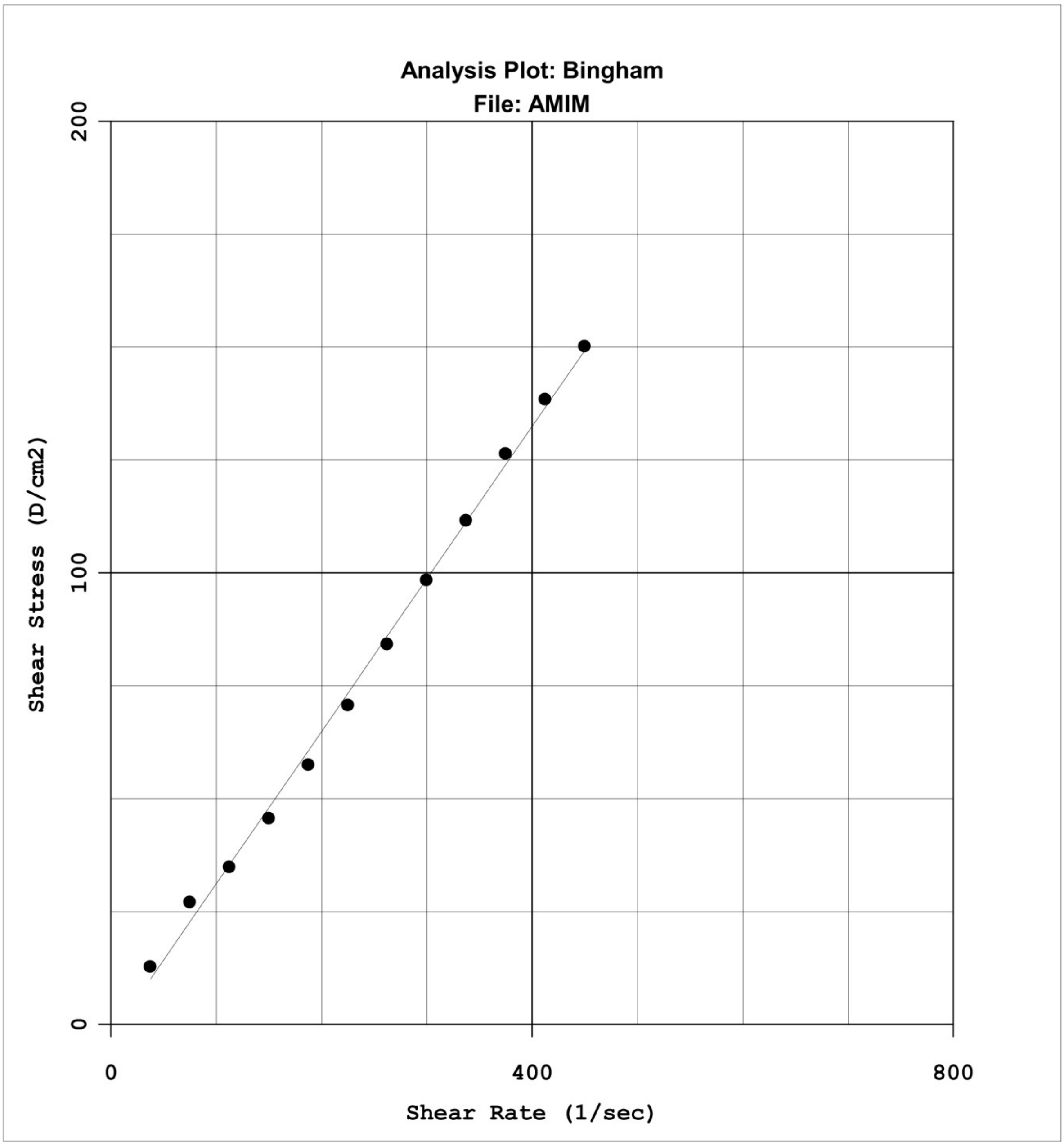

Plastic Viscosity = 33.7

Yield Stress = -2.51

CoF = 95.0

^{1}H & ^{13}C NMR, HR-MS, viscosity of [ButMIm](OTf)

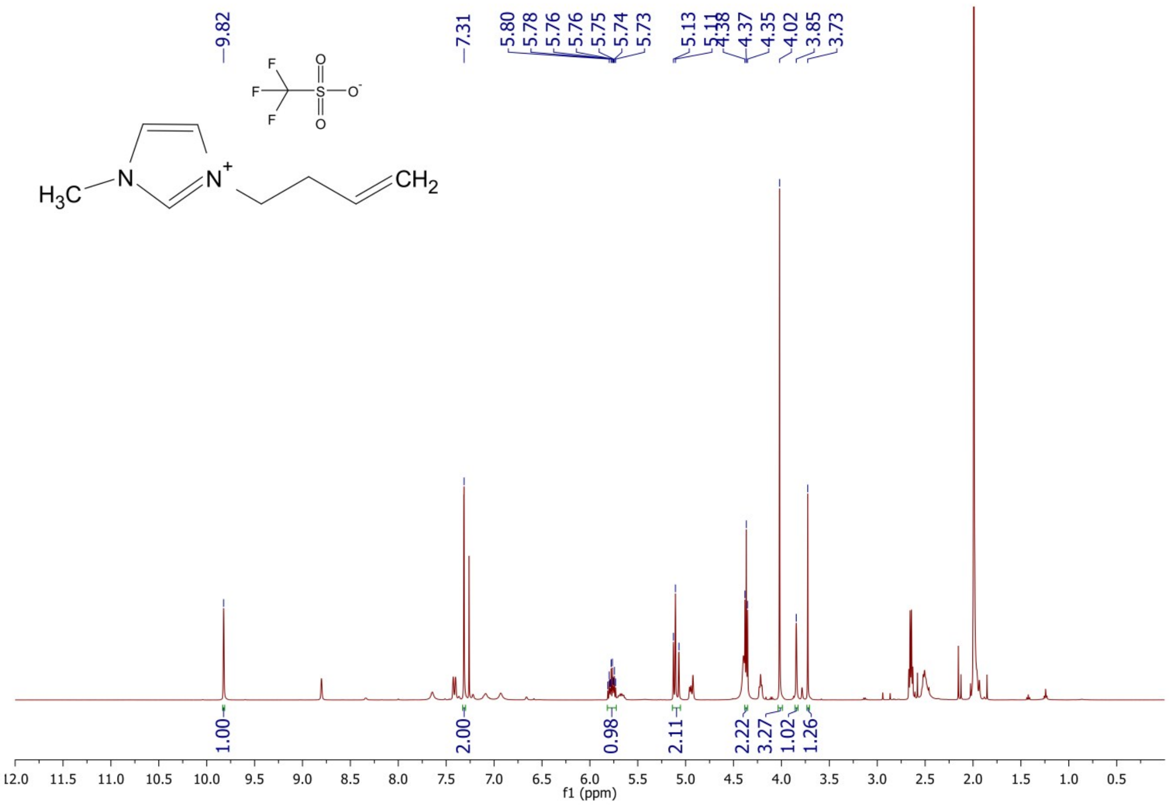

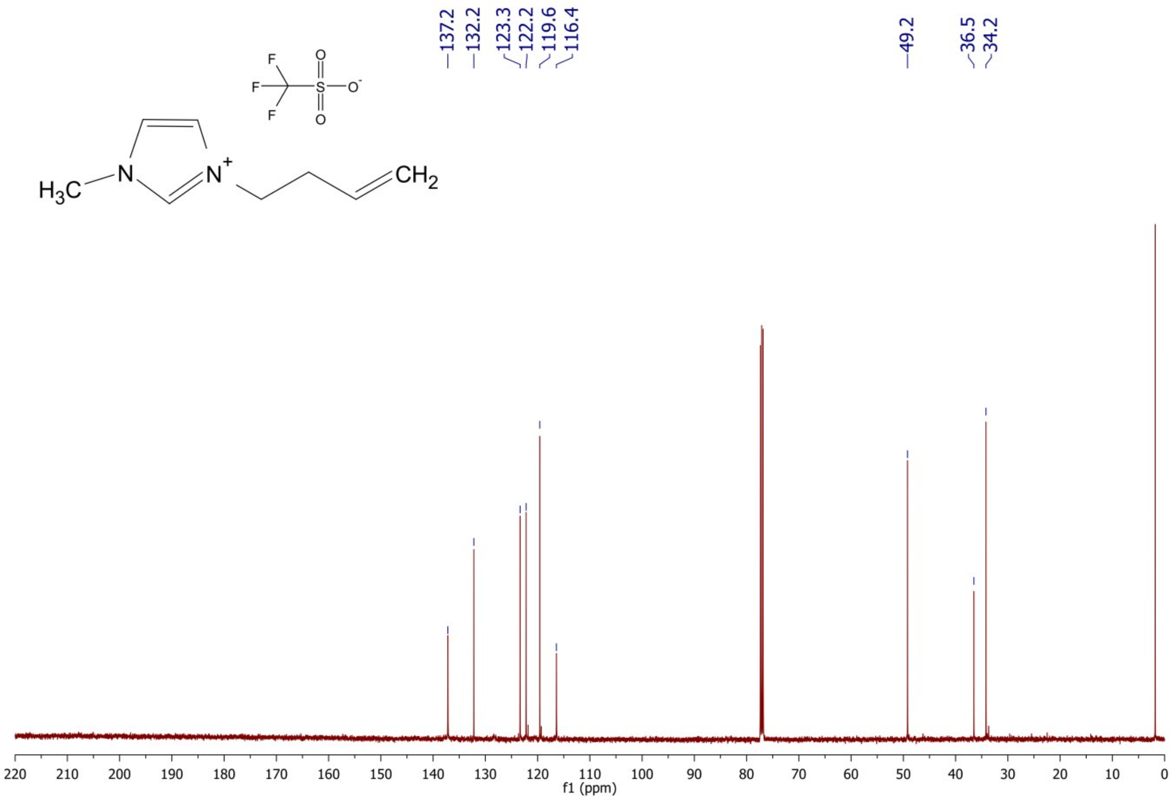

Generated by Foxit PDF Creator © Foxit Software
http://www.foxitsoftware.com For evaluation only.

Display Report

Analysis Info

Analysis Name	D:\Data\dmm 2015\butmim_1-d,1_01_15940.d	Acquisition Date	5/11/2015 11:50:26 AM
Method	dmm.m	Operator	Mai
Sample Name	butmim	Instrument	micrOTOF-Q 10187
Comment			

Acquisition Parameter

Source Type	ESI	Ion Polarity	Positive	Set Nebulizer	1.2 Bar
Focus	Not active	Set Capillary	4500 V	Set Dry Heater	200 °C
Scan Begin	50 m/z	Set End Plate Offset	-500 V	Set Dry Gas	9.0 l/min
Scan End	500 m/z	Set Collision Cell RF	200.0 Vpp	Set Divert Valve	Source

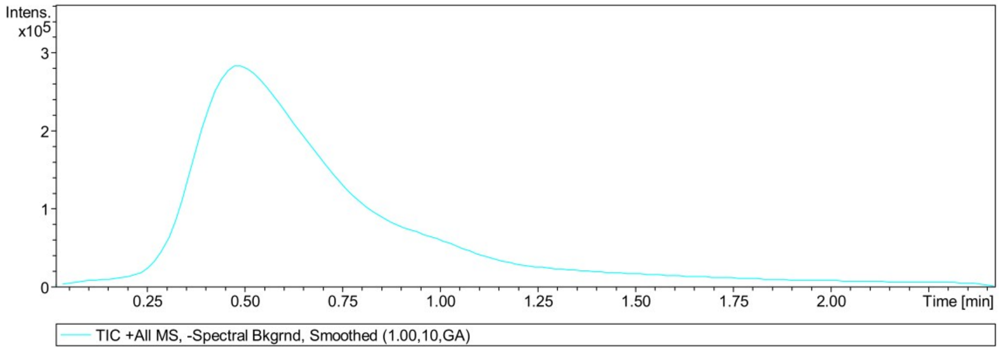

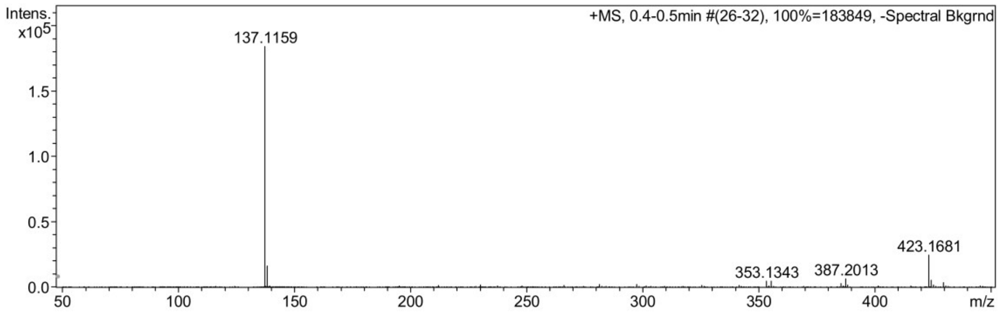

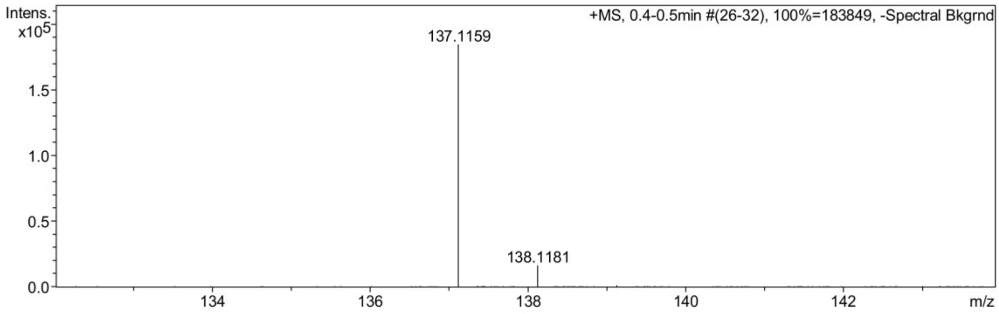

**Rheocalc V2.7**	**Brookfield Engineering Labs**

Math Model: Bingham

Plastic Viscosity: 16,9 cP **Yield Stress: 0,55 D/cm²**

Confidence of Fit: 96,3 %

File: F:\24-6-15\BUTMIM.DB

Test Date: 25/06/2015 **Test Time:** 7:27:30 SA **Model:** RV **Spindle:** CP40

Sample Name: ButMim

#	Viscosity (cP)	Speed (RPM)	% Torque (%)	Shear Stress (D/cm²)	Shear Rate (1/sec)	Temperature (°C)	Time Interval (mm:ss.t)
1	15,04	5,00	2,3	5,64	37,50	29,7	00:30,
2	19,29	10,00	5,9	14,47	75,00	29,8	00:30,
3	16,79	15,00	7,7	18,88	112,50	29,8	00:30,
4	16,02	20,00	9,8	24,03	150,00	29,7	00:30,
5	17,13	25,00	13,1	32,13	187,50	29,8	00:30,
6	17,66	30,00	16,2	39,73	225,00	29,9	00:30,
7	17,56	35,00	18,8	46,11	262,50	30,0	00:30,
8	17,58	40,00	21,5	52,73	300,00	30,0	00:30,
9	17,44	45,00	24,0	58,86	337,50	30,0	00:30,
10	17,07	50,00	26,1	64,01	375,00	30,0	00:30,
11	17,06	55,00	28,7	70,39	412,50	30,0	00:30,
12	17,00	60,00	31,2	76,52	450,00	30,0	00:30,
13	17,00	65,00	33,8	82,89	487,50	30,0	00:30,
14	17,00	70,00	36,4	89,27	525,00	30,0	00:30,
15	16,83	75,00	38,6	94,67	562,50	30,0	00:30,

Notes:

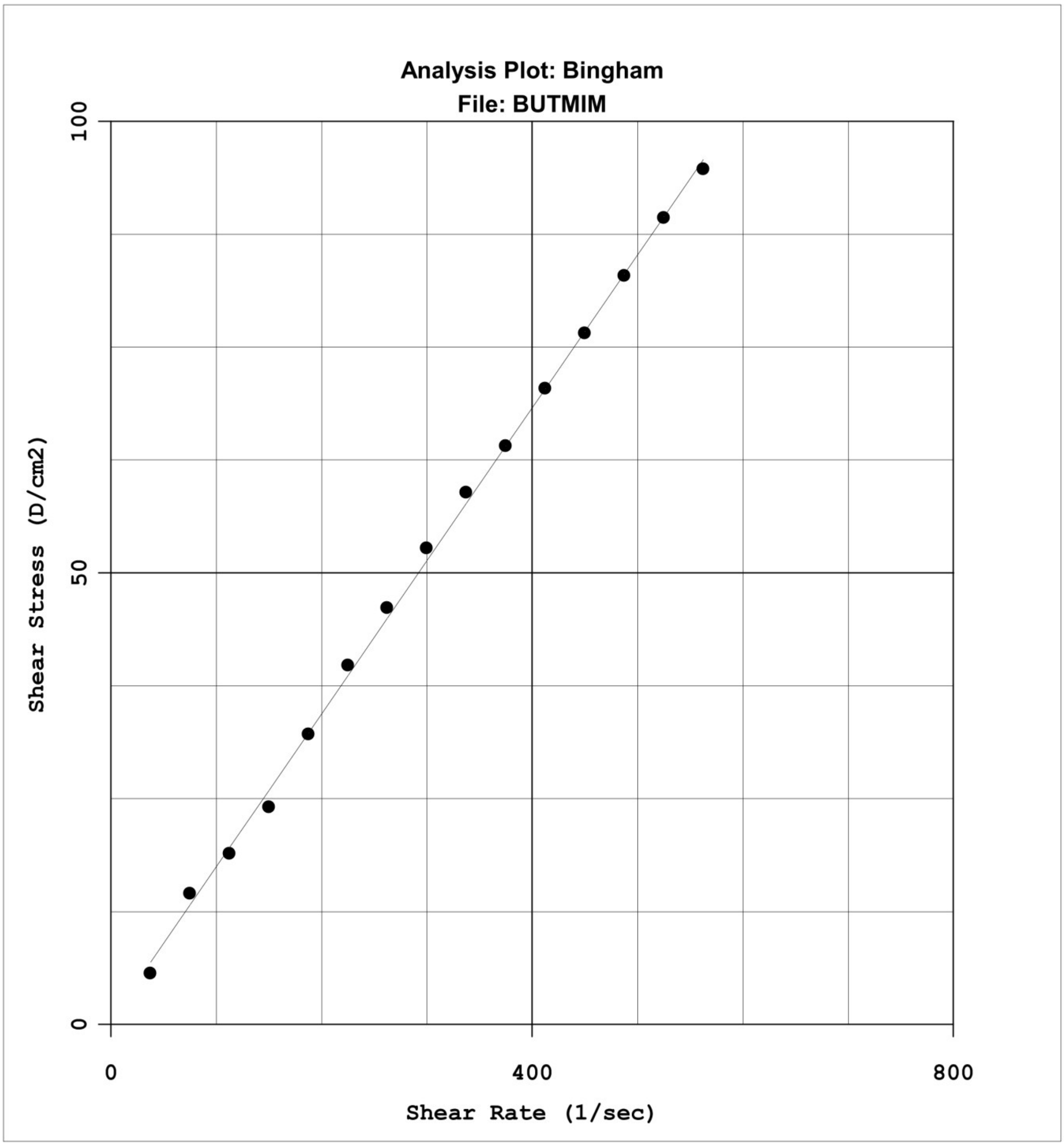

Plastic Viscosity = 16,9

Yield Stress = 0,55

CoF = 96,3

^{1}H & ^{13}C NMR, HR-MS, viscosity of [PentMIm]OTf

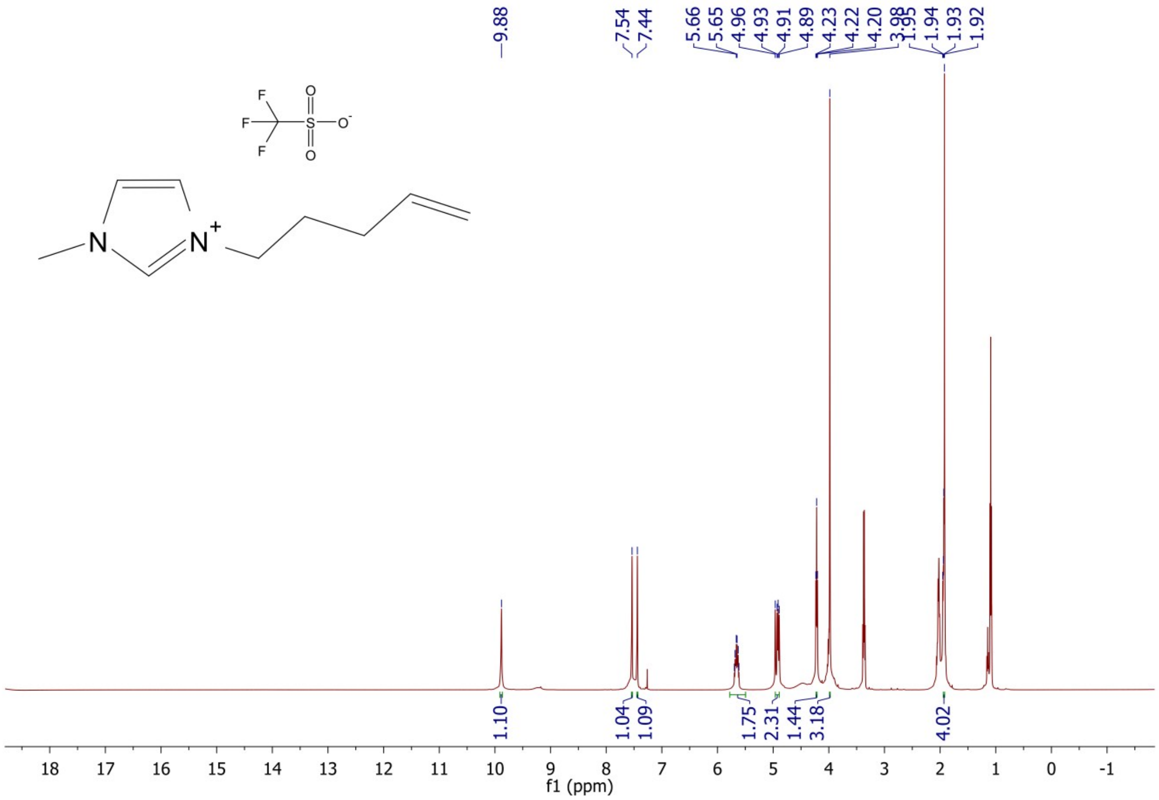

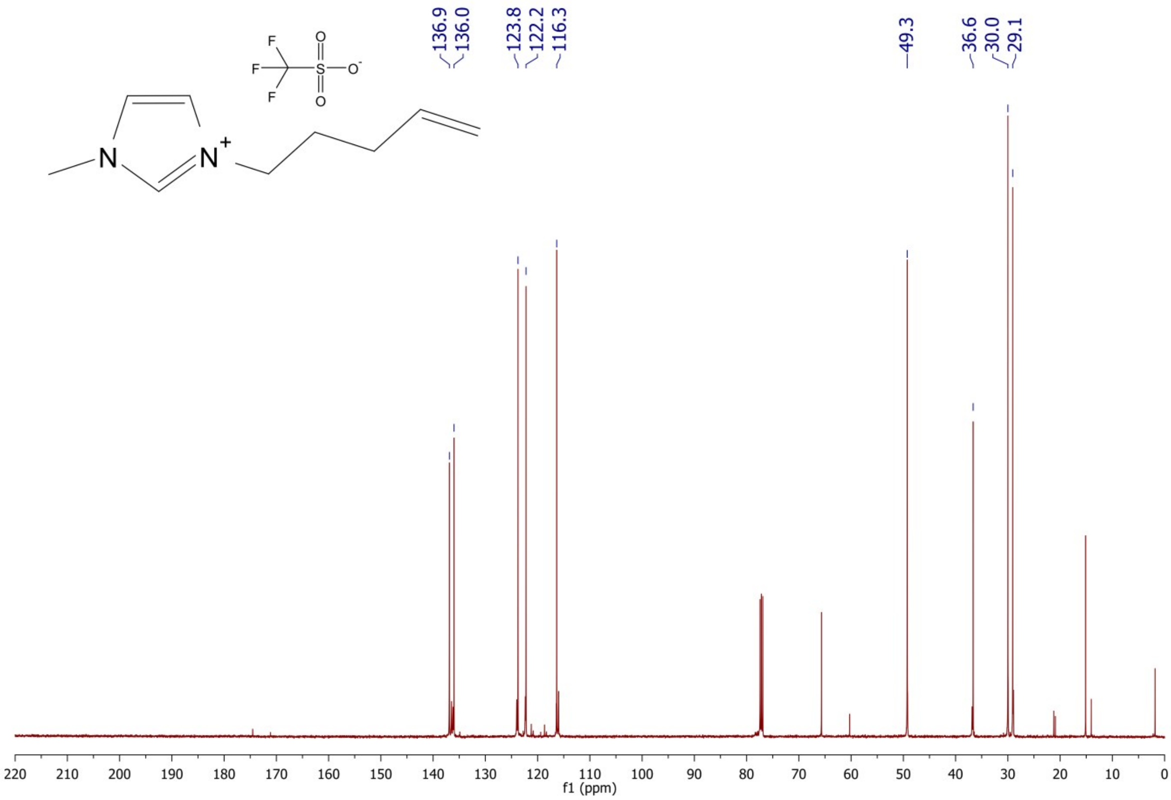

Generated by Foxit PDF Creator © Foxit Software
http://www.foxitsoftware.com For evaluation only.

Display Report

Analysis Info

Analysis Name	D:\Data\dmm 2015\pent mim_1-c,1_01_14978.d	Acquisition Date	3/20/2015 5:35:29 PM
Method	protein my mass tb.m	Operator	Mai
Sample Name	pent mim	Instrument	micrOTOF-Q 10187
Comment			

Acquisition Parameter

Source Type	ESI	Ion Polarity	Positive	Set Nebulizer	1.2 Bar
Focus	Not active	Set Capillary	4500 V	Set Dry Heater	200 °C
Scan Begin	50 m/z	Set End Plate Offset	-500 V	Set Dry Gas	8.0 l/min
Scan End	3000 m/z	Set Collision Cell RF	120.0 Vpp	Set Divert Valve	Source

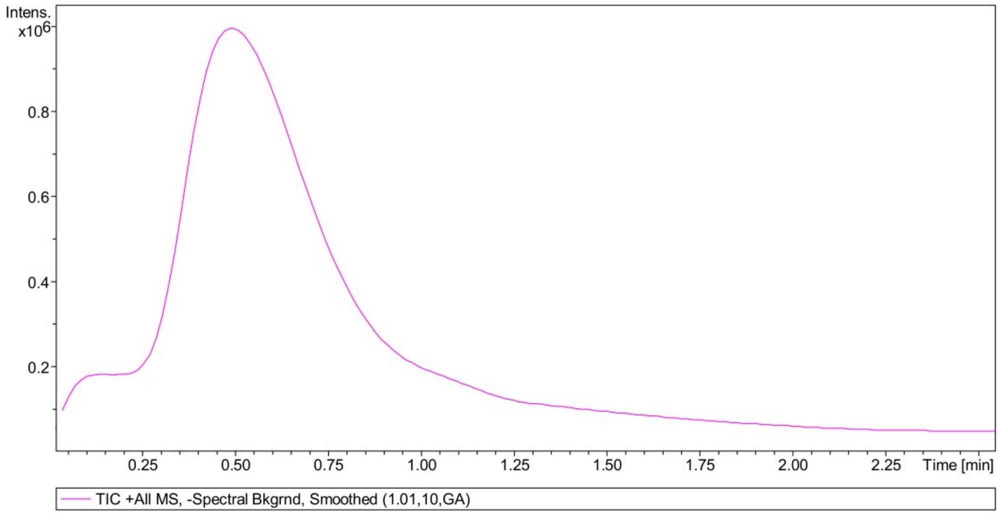

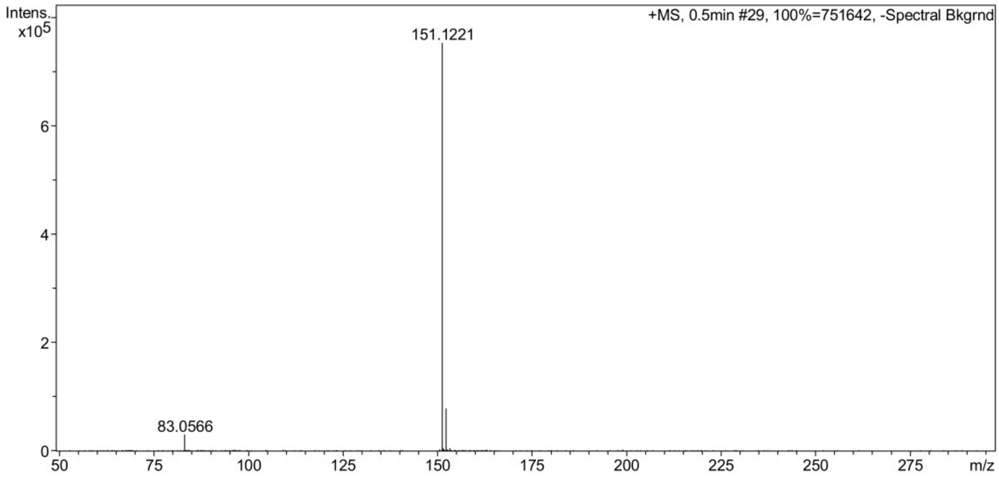

Rheocalc V2.7 **Brookfield Engineering Labs**

Math Model: Bingham

Plastic Viscosity: 94.4 cP **Yield Stress: 1.10 D/cm²**

Confidence of Fit: 98.2 %

File: D:\Do do nhot\Truong Hai (KHTN)\Penmim.DB

Test Date: 3/12/2015 **Test Time:** 2:04:06 PM **Model:** RV **Spindle:** CP40

Sample Name: Penmim

#	Viscosity (cP)	Speed (RPM)	% Torque (%)	Shear Stress (D/cm²)	Shear Rate (1/sec)	Temperature (°C)	Time Interval (mm:ss.t)
1	92.21	5.00	14.1	34.58	37.50	31.3	00:30.2
2	96.47	10.00	29.5	72.35	75.00	31.3	00:30.2
3	97.45	15.00	44.7	109.63	112.50	31.2	00:30.2
4	94.83	20.00	58.0	142.25	150.00	31.1	00:30.2
5	95.88	25.00	73.3	179.77	187.50	31.1	00:30.2
6	93.96	30.00	86.2	211.41	225.00	31.1	00:30.2

Notes:

Page 1 **3/12/2015 2:09:45 PM**

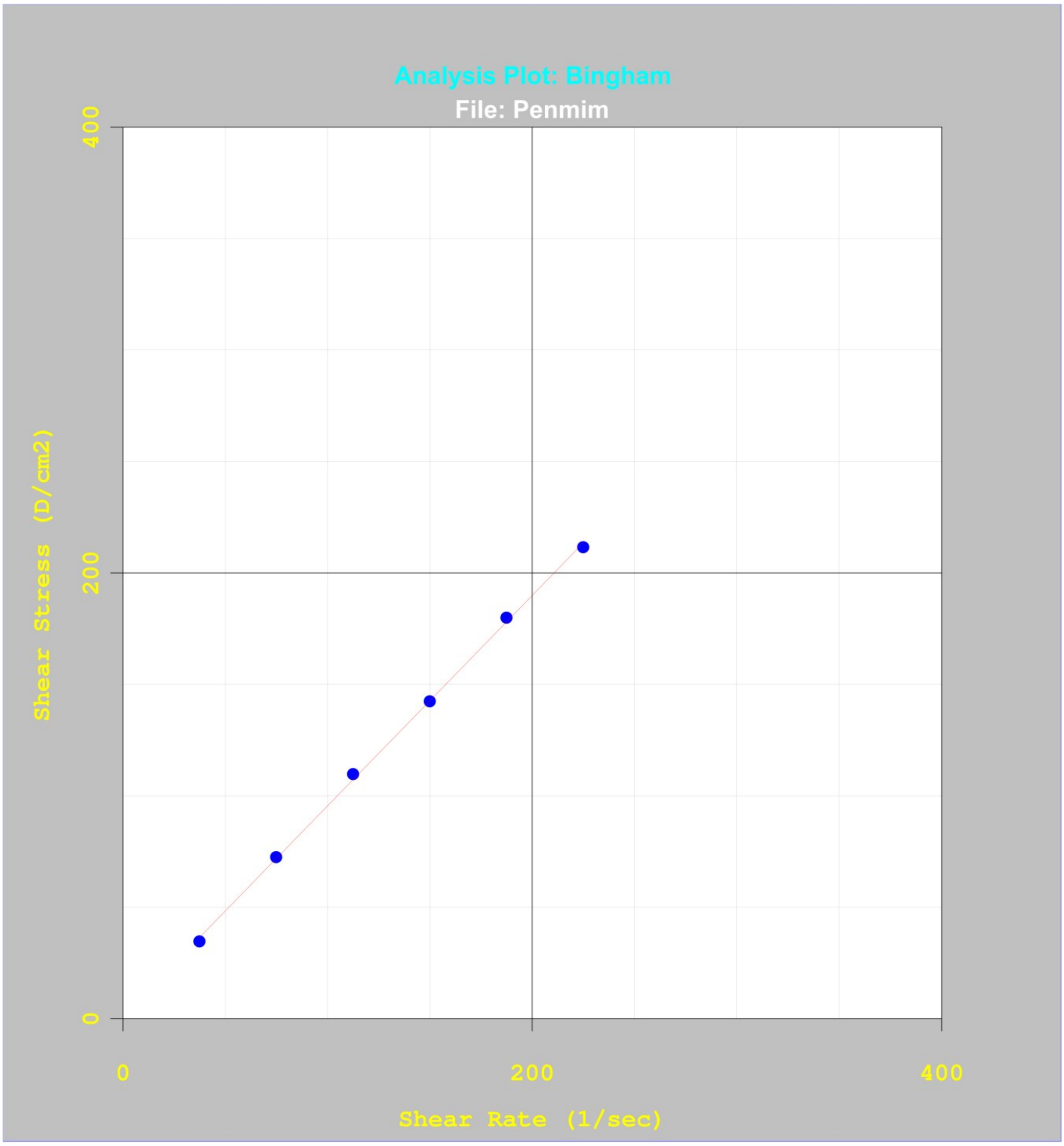

Plastic Viscosity = 94.4

Yield Stress = 1.10

CoF = 98.2

References

1. N. Papageorgiou, *J. Electrochem. Soc.*, 1996, **143**, 3099-3108.
2. H. Matsumoto, T. Matsuda, T. Tsuda, R. Hagiwara, Y. Ito and Y. Miyazaki, *Chem. Lett.*, 2001, **30**, 26-27.
3. P. Wang, S. M. Zakeeruddin, J.-E. Moser and M. Grätzel, *J. Physic. Chem. B*, 2003, **107**, 13280-13285.
4. P. Wang, S. M. Zakeeruddin, R. Humphry-Baker and M. Grätzel, *Chem. Mater.*, 2004, **16**, 2694-2696.
5. R. Kawano, H. Matsui, C. Matsuyama, A. Sato, M. A. B. H. Susan, N. Tanabe and M. Watanabe, *J. Photochem. Photobiol. A: Chem.*, 2004, **164**, 87-92.
6. P. Wang, B. Wenger, R. Humphry-Baker, J.-E. Moser, J. Teuscher, W. Kantlehner, J. Mezger, E. V. Stoyanov, S. M. Zakeeruddin and M. Grätzel, *J. Am. Chem. Soc.*, 2005, **127**, 6850-6856.
7. D. Kuang, P. Wang, S. Ito, S. M. Zakeeruddin and M. Grätzel, *J. Am. Chem. Soc.*, 2006, **128**, 7732-7733.
8. D. Kuang, C. Klein, Z. Zhang, S. Ito, J. E. Moser, S. M. Zakeeruddin and M. Gratzel, *Small*, 2007, **3**, 2094-2102.
9. M. Gorlov, H. Pettersson, A. Hagfeldt and L. Kloo, *Inorg. Chem.*, 2007, **46**, 3566-3575.
10. Y. Wang, Y. Sun, B. Song and J. Xi, *Sol. Energy Mater. Sol. Cells*, 2008, **92**, 660-666.
11. H. Gamstedt, A. Hagfeldt and L. Kloo, *Polyhedron*, 2009, **28**, 757-762.
12. P. Cheng, W. Wang, T. Lan, R. Chen, J. Wang, J. Yu, H. Wu, H. Yang, C. Deng and S. Guo, *J. Photochem. Photobiol. A: Chem.*, 2010, **212**, 147-152.
13. L. Guo, X. Pan, M. Wang, C. Zhang, X. Fang, S. Chen and S. Dai, *Solar Energy*, 2011, **85**, 7-11.
14. A. Abate, A. Petrozza, V. Roiati, S. Guarnera, H. Snaith, F. Matteucci, G. Lanzani, P. Metrangolo and G. Resnati, *Organic Electronics*, 2012, **13**, 2474-2478.
15. H. Wu, Z. Lv, S. Hou, X. Cai, D. Wang, H. Kafafy, Y. Fu, C. Zhang, Z. Chu and D. Zou, *J. Power Sources*, 2013, **221**, 328-333.
16. S. M. M. Yusof and W. Z. N. Yahya, *Procedia Engineering*, 2016, **148**, 100-105.
17. S. Denizalti, A. K. Ali, Ç. Ela, M. Ekmekci and S. Erten-Ela, *Chem. Phys. Lett.*, 2018, **691**, 373-378.
